# Supplementary material for: Antrodia Cinnamomea Prolongs Survival in a Patient with Small Cell Lung Cancer
Source: Medicina (Kaunas). 2019 Sep 26;55(10):640. doi: 10.3390/medicina55100640 (PMC6843373; doi:10.3390/medicina55100640)
Supplement: Supplementary file 1 [file medicina-55-00640-s001.zip › medicina-564676-suppls/Table S2.docx]

| **Table S2 - the patient's fasting blood glucose and glycated haemoglobin levels in the past two years without DAC treatment** | | |
| --- | --- | --- |
| **Date** | **HbA1c** | **GAC** |
| **18-Mar-14** | 7.2 | 124 |
| **17-Jun-14** | 7 | 122 |
| **16-Sep-14** | 6.9 | NA |
| **10-Dec-14** | 6.6 | 115 |
| **17-Mar-15** | 7.6 | 127 |
| **16-Jun-15** | 6.8 | 107 |
| **13-Sep-15** | 6.7 | NA |
| **16-Dec-15** | **7.2** | **140** |
| **12-Mar-16*** | **6.8** | **109** |
| **7-Sep-16*** | **6.2** | **94** |
| *The dates during or after the period of DAC treatment.  HbA1c = Glycated haemoglobin; GAC = Glucose antecibum (before meals) | | |
