# Supplementary figures and images for: Antrodia Cinnamomea Prolongs Survival in a Patient with Small Cell Lung Cancer
Source: Medicina (Kaunas). 2019 Sep 26;55(10):640. doi: 10.3390/medicina55100640 (PMC6843373; doi:10.3390/medicina55100640)

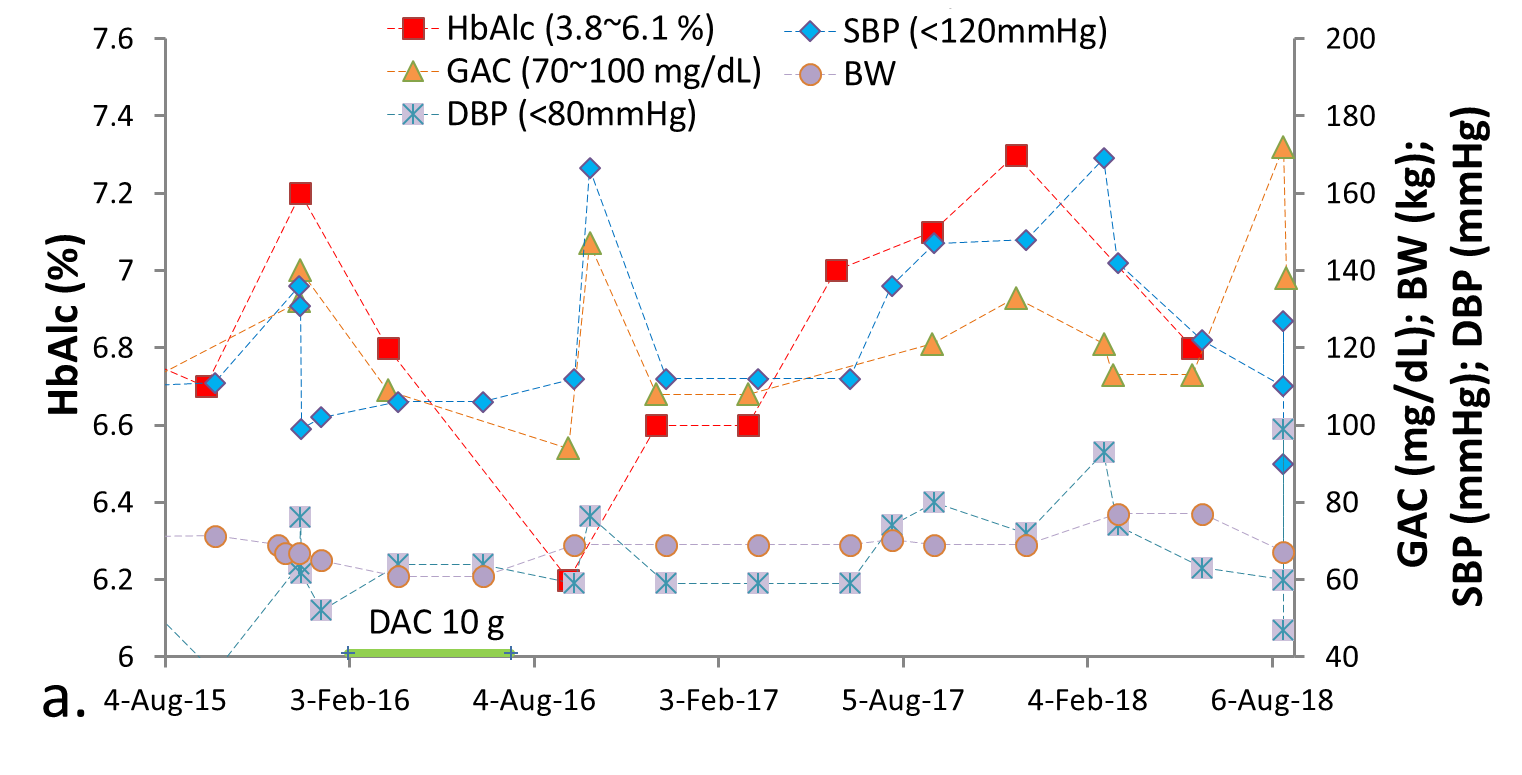

Supplement: Supplementary file 1 [file medicina-55-00640-s001.zip › Figure S1 a.png]

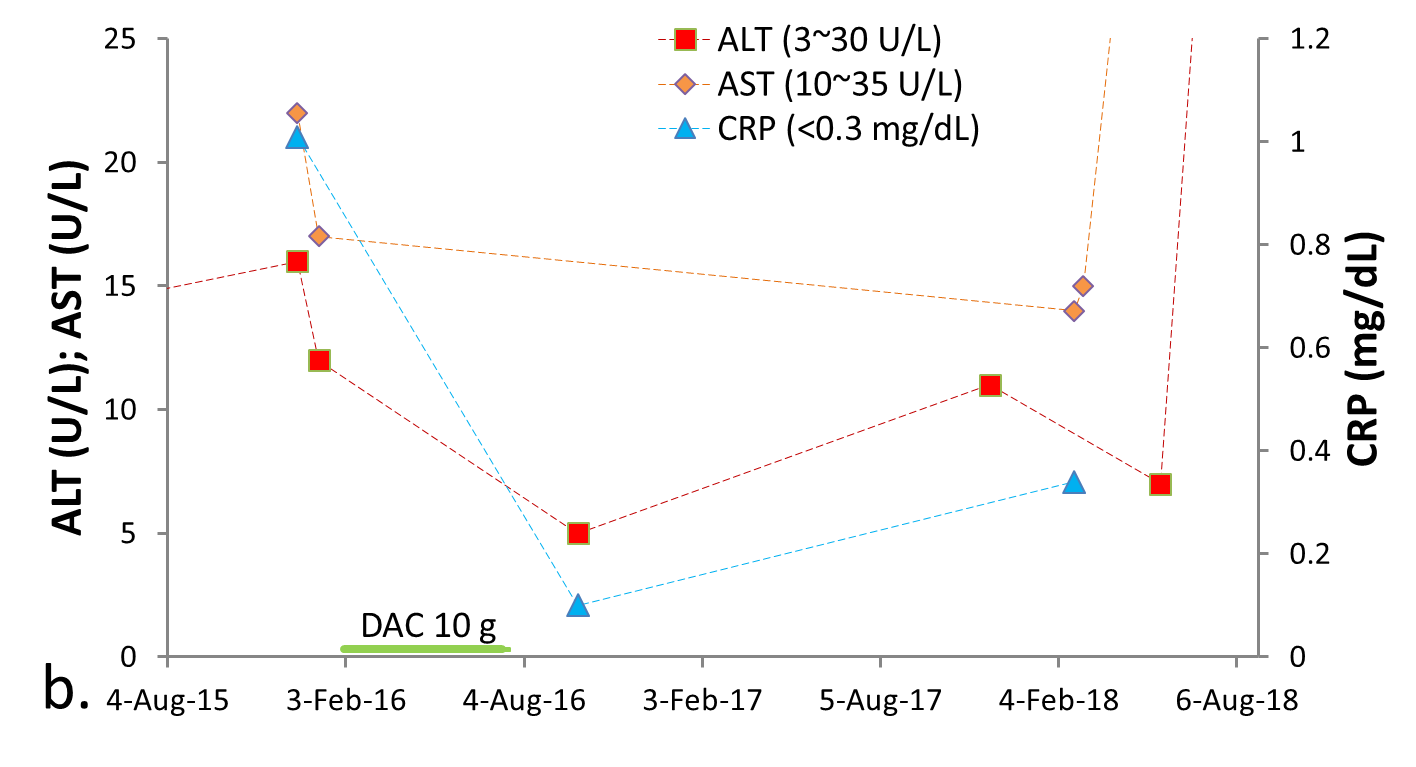

Supplement: Supplementary file 1 [file medicina-55-00640-s001.zip › Figure S1 b.png]

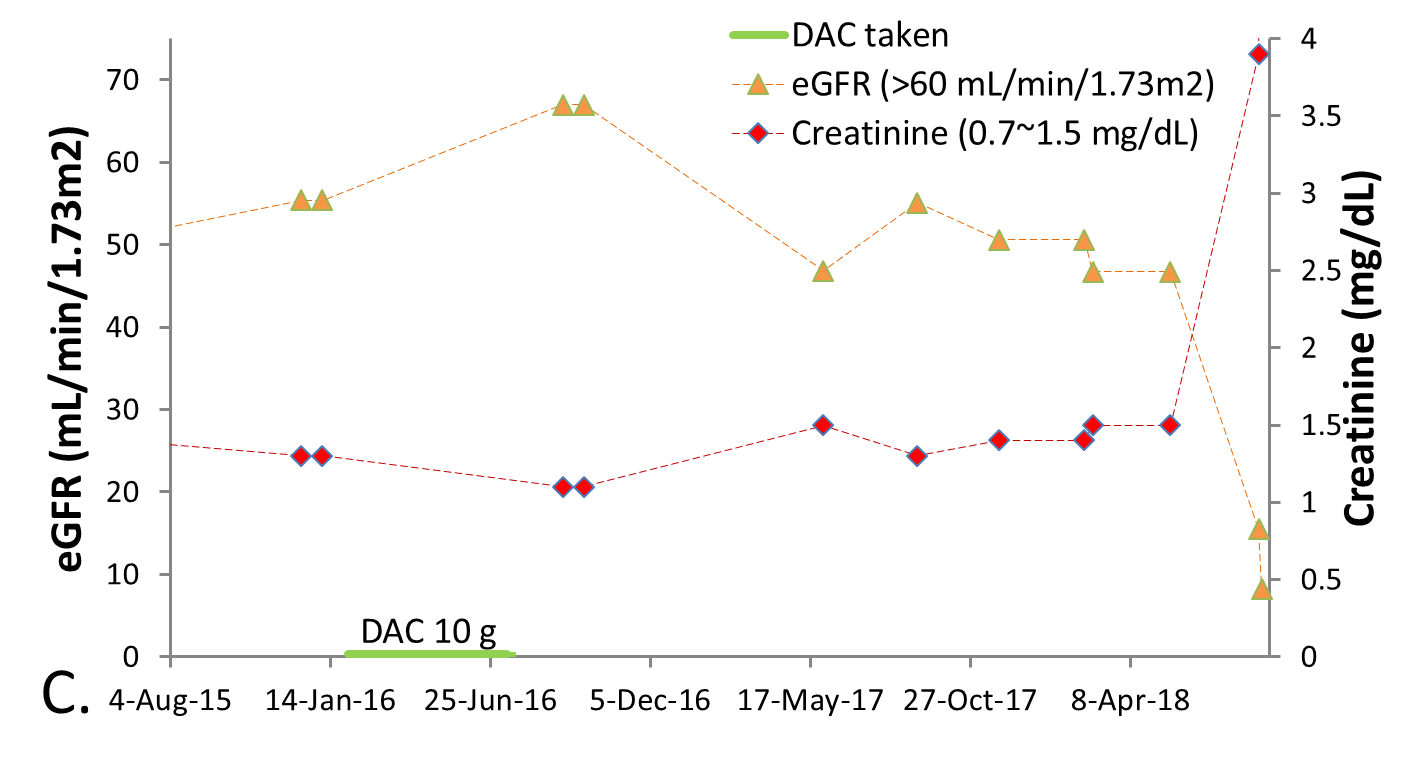

Supplement: Supplementary file 1 [file medicina-55-00640-s001.zip › Figure S1 c.png]

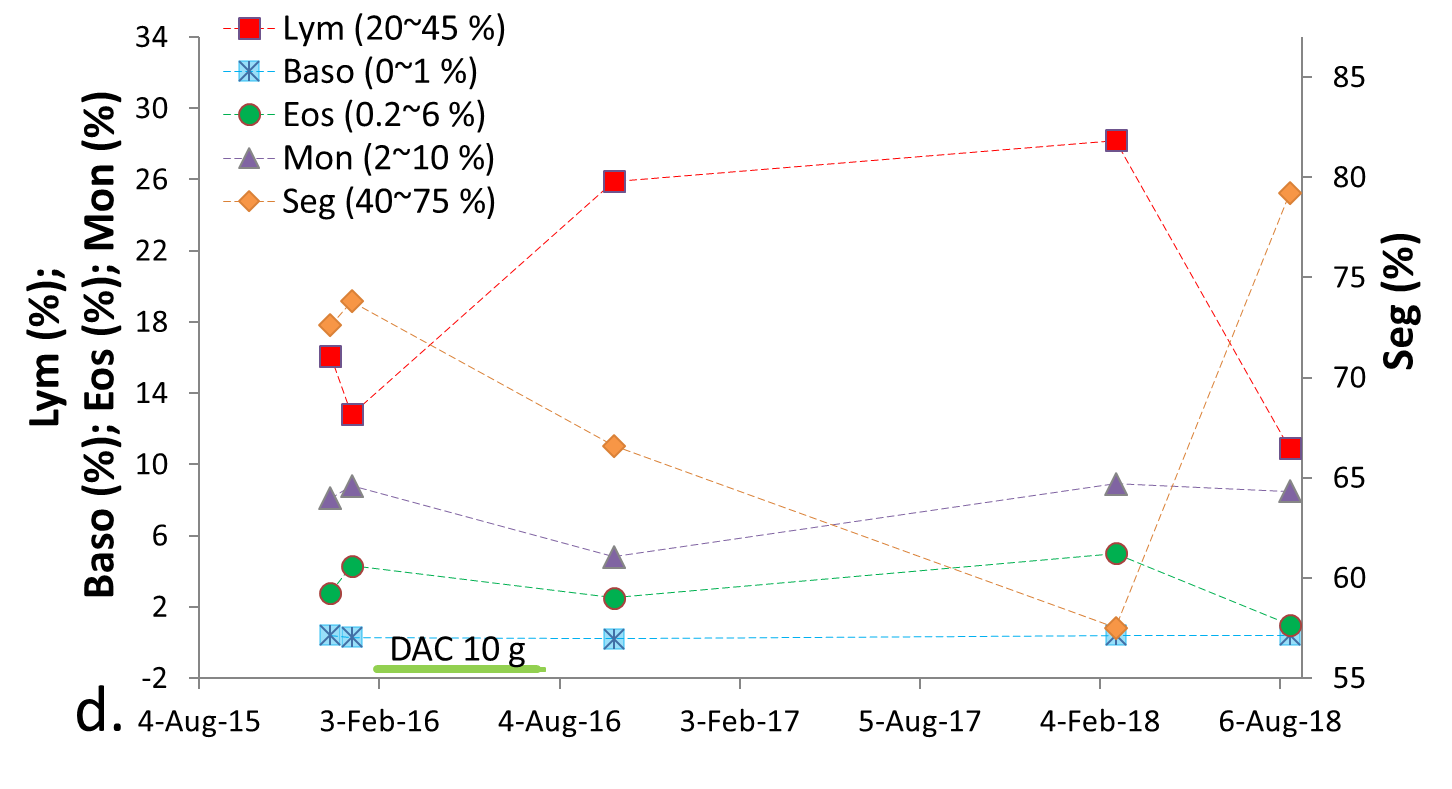

Supplement: Supplementary file 1 [file medicina-55-00640-s001.zip › Figure S1 d.png]

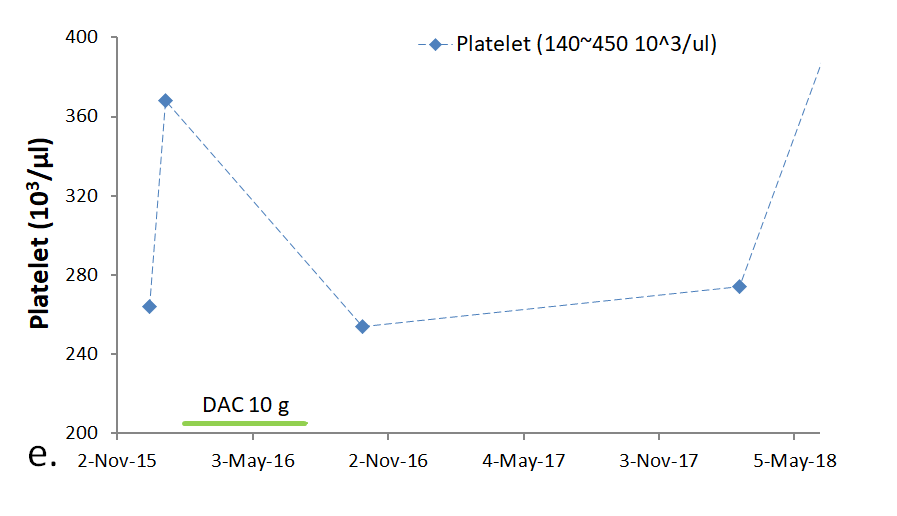

Supplement: Supplementary file 1 [file medicina-55-00640-s001.zip › Figure S1 e.png]

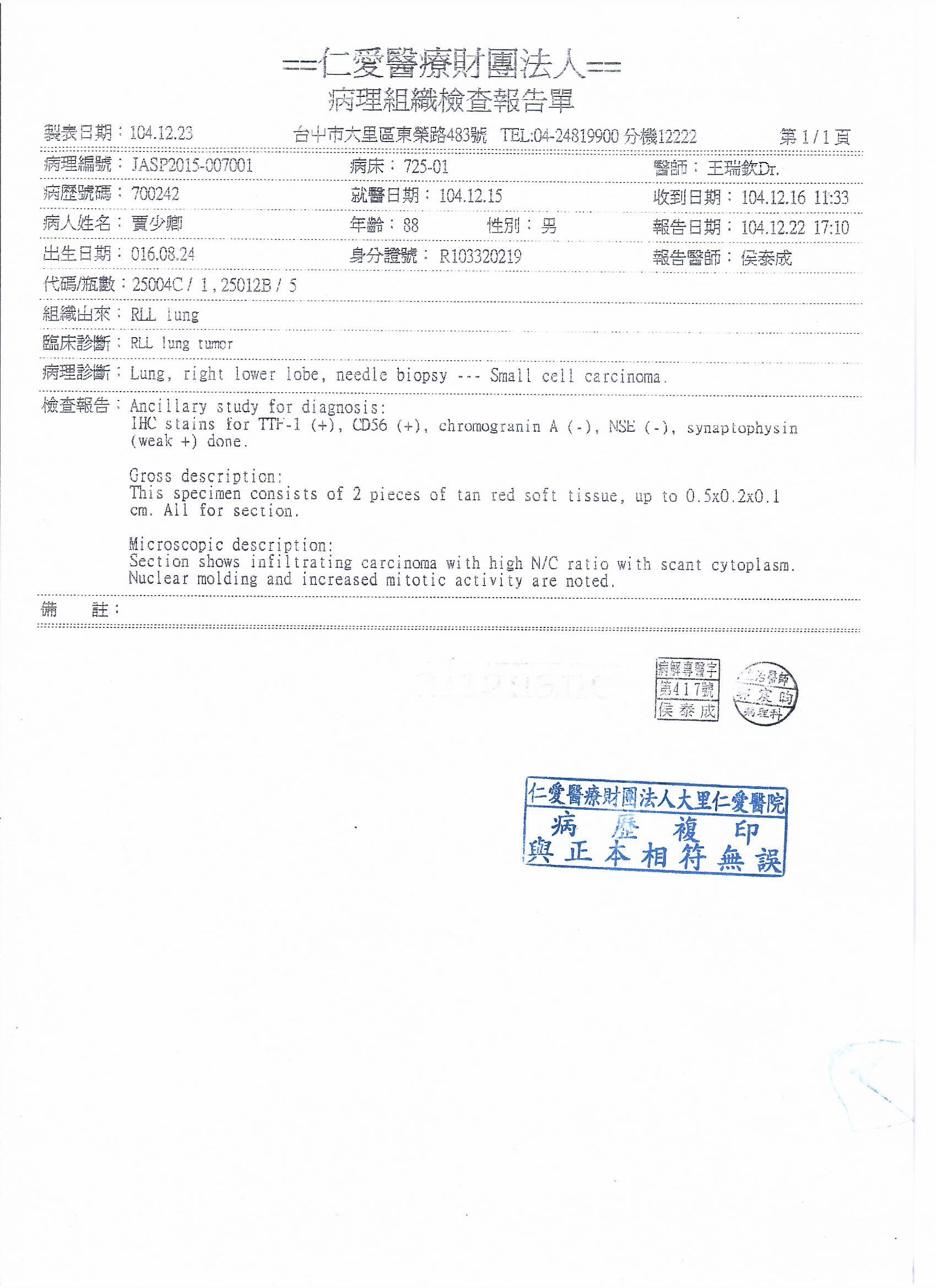

Supplement: Supplementary file 1 [file medicina-55-00640-s001.zip › medicina-564676-suppls/Table S1.jpg]
